# Supplementary material for: Glycerol contributes to tuberculosis susceptibility in male mice with type 2 diabetes
Source: Nat Commun. 2023 Sep 20;14:5840. doi: 10.1038/s41467-023-41519-9 (PMC10511404; doi:10.1038/s41467-023-41519-9)
Supplement: Supplementary file 1 — Supplementary Information [file 41467_2023_41519_MOESM1_ESM.pdf]

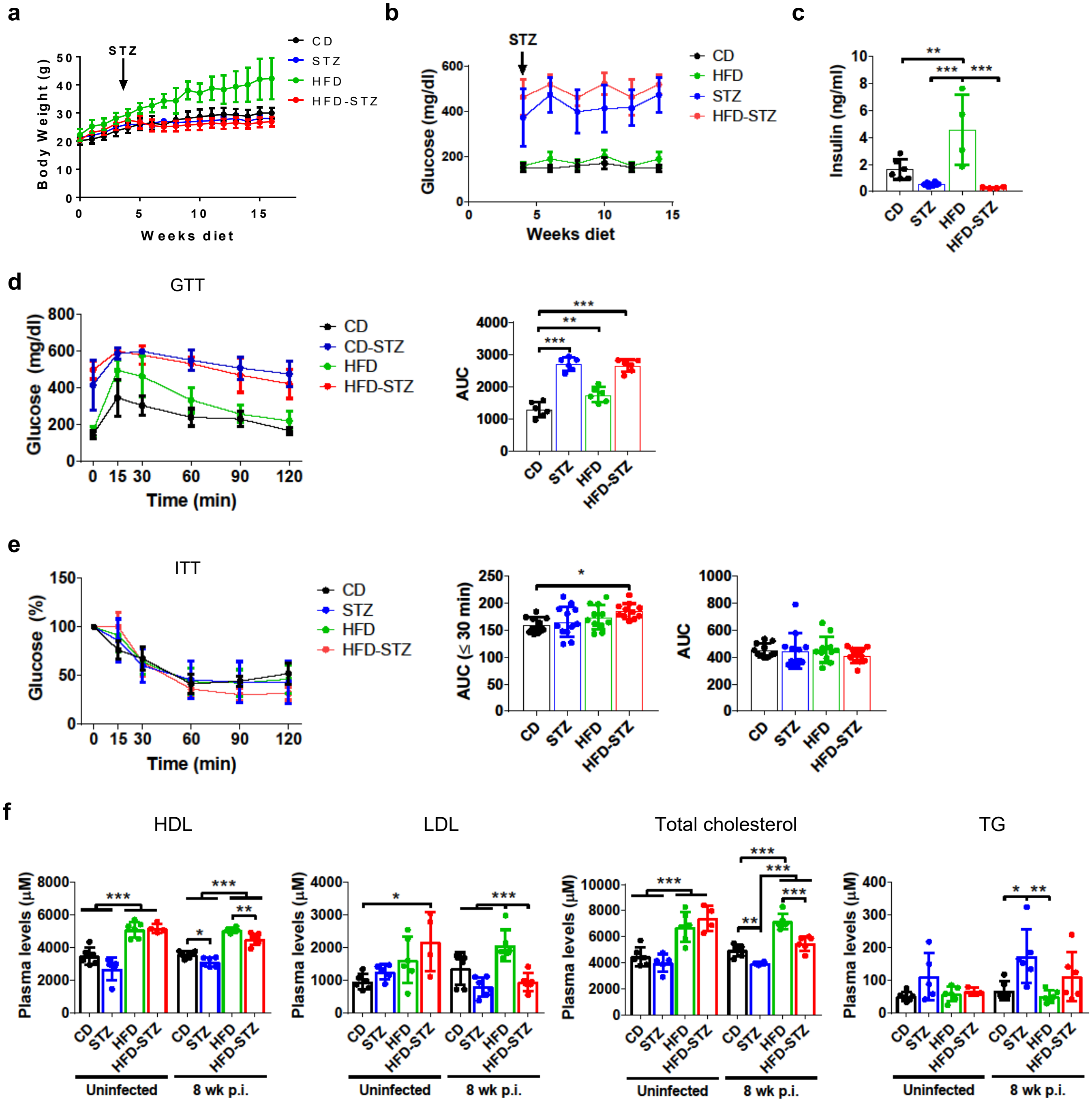
a
STZ
Body Weight (g)
Weeks diet
CD
STZ
HFD
HFD-STZ
b
STZ
Glucose (mg/dl)
Weeks diet
CD
HFD
STZ
HFD-STZ
c
Insulin (ng/ml)
CD
STZ
HFD
HFD-STZ
d
GTT
Glucose (mg/dl)
Time (min)
CD
CD-STZ
HFD
HFD-STZ
AUC
CD
STZ
HFD
HFD-STZ
e
ITT
Glucose (%)
Time (min)
CD
STZ
HFD
HFD-STZ
AUC (≤ 30 min)
AUC
f
HDL
LDL
Total cholesterol
TG
Plasma levels (µM)
Uninfected
8 wk p.i.

Figure S1. Streptozotocin treatment combined with high fat diet mirrors human type 2 diabetes. a Body weight of uninfected mice on a control diet (*CD*), on a high fat diet (*HFD*), treated with streptozotocin (*STZ*) or a combination of both (*HFD-STZ*) (n=6 mice). **b** Non-fasting blood glucose levels in uninfected mice (n=6 mice). **c** Insulin plasma levels from age-matched uninfected mice (n=4 mice for HFD and HFD-STZ and n=6 mice for the others). **d** Glucose tolerance test (GTT, n=6 mice) and **e** insulin tolerance test (ITT, n=12 mice for STZ, n=11 mice for the others) performed in uninfected mice that were age-matched to the infected mice (Fig. 1). Area under the curve (*AUC*) was calculated for the first 30 min for ITT (*middle*) or the total in both GTT and ITT (*right*). **f** Levels of high density lipoprotein (*HDL*), low density lipoprotein (*LDL*), total cholesterol and triglycerides (*TG*) in plasma (n=4 mice for uninfected HFD-STZ, n=5 mice for uninfected STZ and n=6 mice for the others). Data are expressed as mean ± SD. The experiments were repeated at least twice. Statistical analysis was performed by One-Way ANOVA separately for uninfected or 8 wk p.i., *P<0.05, **P<0.01 and ***P<0.001 (*p* value from left to right= **c**: 0.0086, 0.0005, 0.0006; **d**: <0.0001, 0.0067, <0.0001; **e**: 0.0301; **f**: HDL: 0.0003, 0.0006, <0.0001, <0.0001, 0.0405, <0.0001, <0.0001, <0.0001, <0.0001, 0.0070; LDL: 0.0171, 0.0261, 0.0001, 0.0005; Total cholesterol: 0.0002, 0.0005, 0.0005, 0.0005, 0.0002, 0.0082, <0.0001, <0.0001, <0.0001, <0.0001; TG: 0.0232, 0.0074). Source data are provided as Source Data File.

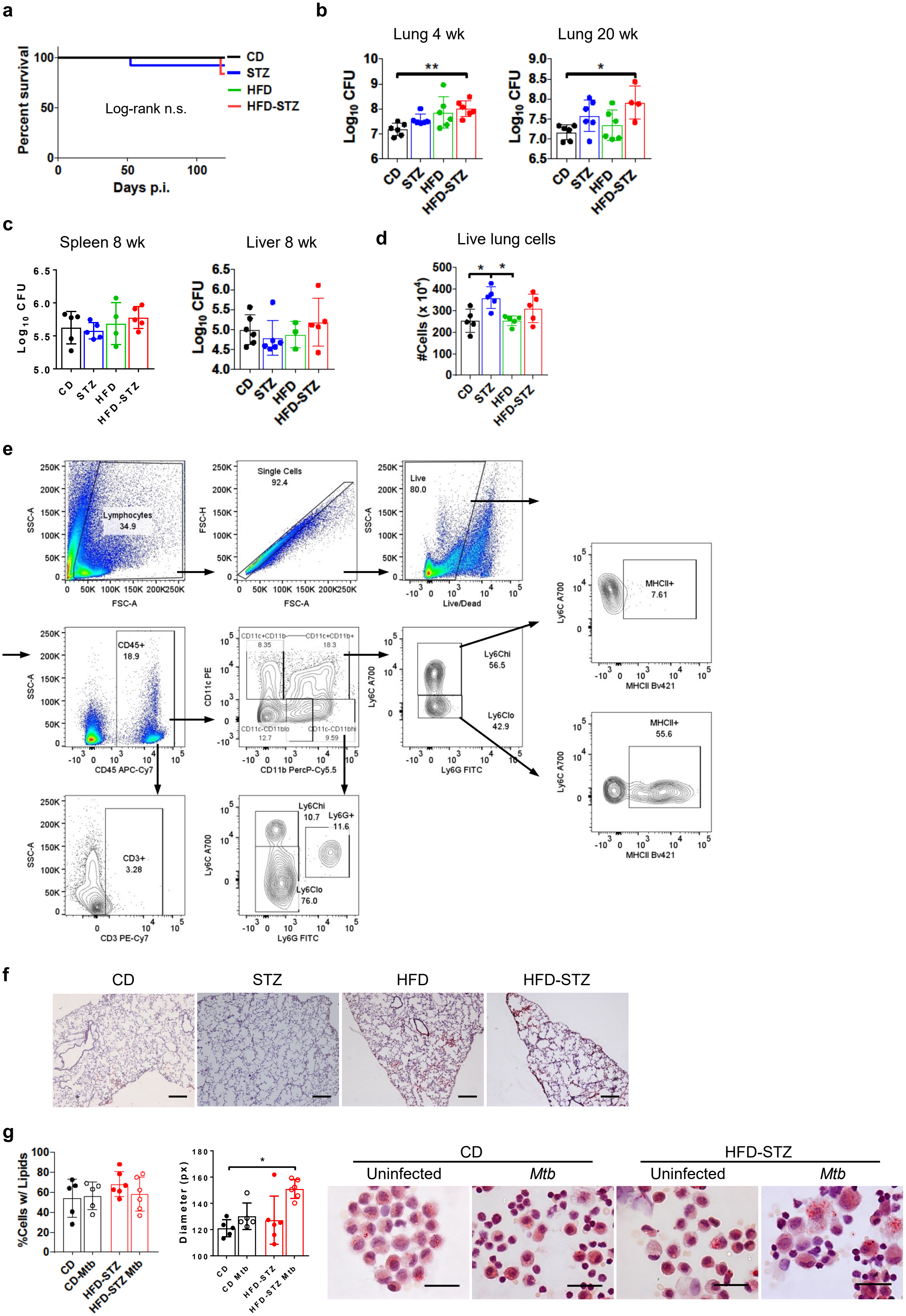
a
Percent survival
Days p.i.
CD
STZ
HFD
HFD-STZ
Log-rank n.s.
b
Lung 4 wk
Lung 20 wk
Log10 CFU
**
*
c
Spleen 8 wk
Liver 8 wk
d
Live lung cells
#Cells (x 10^4)
e
Lymphocytes 34.9
Single Cells 92.4
Live 80.0
SSC-A
FSC-A
FSC-H
Live/Dead
CD45+ 18.9
CD45 APC-Cy7
CD3+ 3.28
CD3 PE-Cy7
CD11c+CD11b- 8.35
CD11c+CD11b+ 18.3
CD11c-CD11blo 12.7
CD11c-CD11bhi 9.59
CD11c PE
CD11b PercP-Cy5.5
Ly6Chi 10.7
Ly6G+ 11.6
Ly6Clo 76.0
Ly6G FITC
Ly6C A700
Ly6Chi 56.5
Ly6Clo 42.9
MHCII+ 7.61
MHCII+ 55.6
MHCII Bv421
f
CD
STZ
HFD
HFD-STZ
g
%Cells w/ Lipids
CD
CD-Mtb
HFD-STZ
HFD-STZ Mtb
Diameter (px)
*
CD
HFD-STZ
Uninfected
Mtb
Uninfected
Mtb

Figure S2. Combination of dyslipidemia and hyperglycemia increases susceptibility to TB. a Survival curve of uninfected untreated mice (*CD*) or mice treated with high fat diet (*HFD*) or streptozotocin (*STZ*) or a combination of both *(HFD-STZ*). Analysis was performed with log-rank Mantel-Cox test (n=13 mice). **b** Bacterial growth in lungs at 4 (n=6 mice) and 20 weeks (n=4 mice for HFD-STZ and n=6 mice for the others) after *Mtb* infection. In the 20-week time point, 2 out of 4 HFD-STZ mice had to be sacrificed at 18 weeks p.i. **c** Bacterial growth in spleen (n=4 mice for HFD and n=5 mice for the others) and liver (n=3 mice for HFD, n=5 mice for HFD-STZ and n=6 mice for the others) at 8 weeks after *Mtb* infection. Data are expressed as mean ± SD. **d** Total number of live cells in the lung (n=5 mice). **e** Gating strategy for flow cytometry staining of lung myeloid cells and T cells. **f** Oil-red-O staining of lung sections from uninfected mice. Scale bar, 100 µm. **g** Bronchoalveolar lavage cells were isolated from uninfected or infected CD and HFD-STZ mice and oil-red-O stained. Percentage of cells that contained lipids and cell diameter were calculated (*left*) (n=5 mice for CD and CD-Mtb and n=6 mice for the others). Representative images taken at 40X (*right*). Scale bar, 500 µm. Statistical analysis was performed by One-Way ANOVA, *P<0.05, **P<0.01 and ***P<0.001 (*p* value from left to right= **b**: 0.0074; **c**: 0.0195; **d**: 0.0193, 0.0204; **g**: 0.0166). Source data are provided as Source Data File.

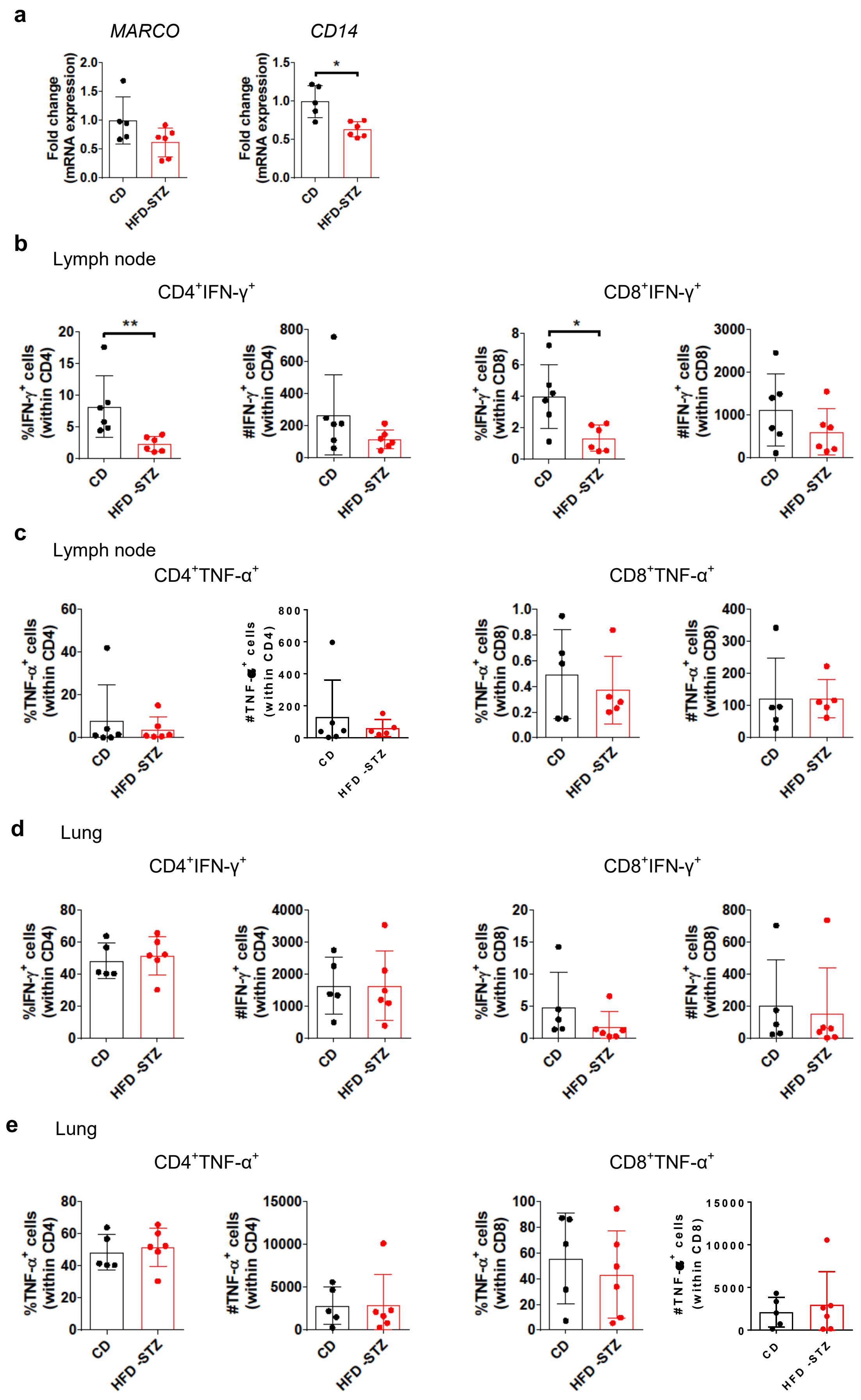
a
MARCO
CD14
Fold change (mRNA expression)
CD
HFD-STZ
*
b
Lymph node
CD4+IFN-γ+
CD8+IFN-γ+
%IFN-γ+ cells (within CD4)
#IFN-γ+ cells (within CD4)
%IFN-γ+ cells (within CD8)
#IFN-γ+ cells (within CD8)
**
*
c
Lymph node
CD4+TNF-α+
CD8+TNF-α+
%TNF-α+ cells (within CD4)
#TNF-α+ cells (within CD4)
%TNF-α+ cells (within CD8)
#TNF-α+ cells (within CD8)
d
Lung
CD4+IFN-γ+
CD8+IFN-γ+
e
Lung
CD4+TNF-α+
CD8+TNF-α+
CD
HFD-STZ

Figure S3. Mice on a high fat diet and treated on streptozotocin have defective innate and adaptive immune responses. a Bronchoalveolar lavage cells were isolated from uninfected control diet mice (*CD*) or mice on a high fat diet and treated with streptozotocin (*HFD-STZ*). Gene expression of *Marco* and *Cd14* measured by RT-qPCR are expressed as fold-change to the control group (n=5 mice for CD and n=6 mice for HFD-STZ). **b** Percentage and number of IFN-γ^+ and **c** TNF-α^+ $CD4^+$ and $CD8^+$ T cells in lung-draining lymph nodes 14 days after aerosol infection with ~100 CFU *Mtb* Erdman (n=6 mice). **d** Percentage and number of IFN-γ^+ and **e** TNF-α^+ $CD4^+$ and $CD8^+$ T cells in lungs 14 days after infection (n=5 mice for CD and n=6 mice for HFD-STZ). Data are expressed as mean ± SD. The experiments were repeated at least twice. Statistical analysis was performed by two-tail Student's t-test, *P<0.05 and **P<0.01 (*p* value from left to right= **a**: 0.0173; **b**: 0.0022, 0.0152). Source data are provided as Source Data File.

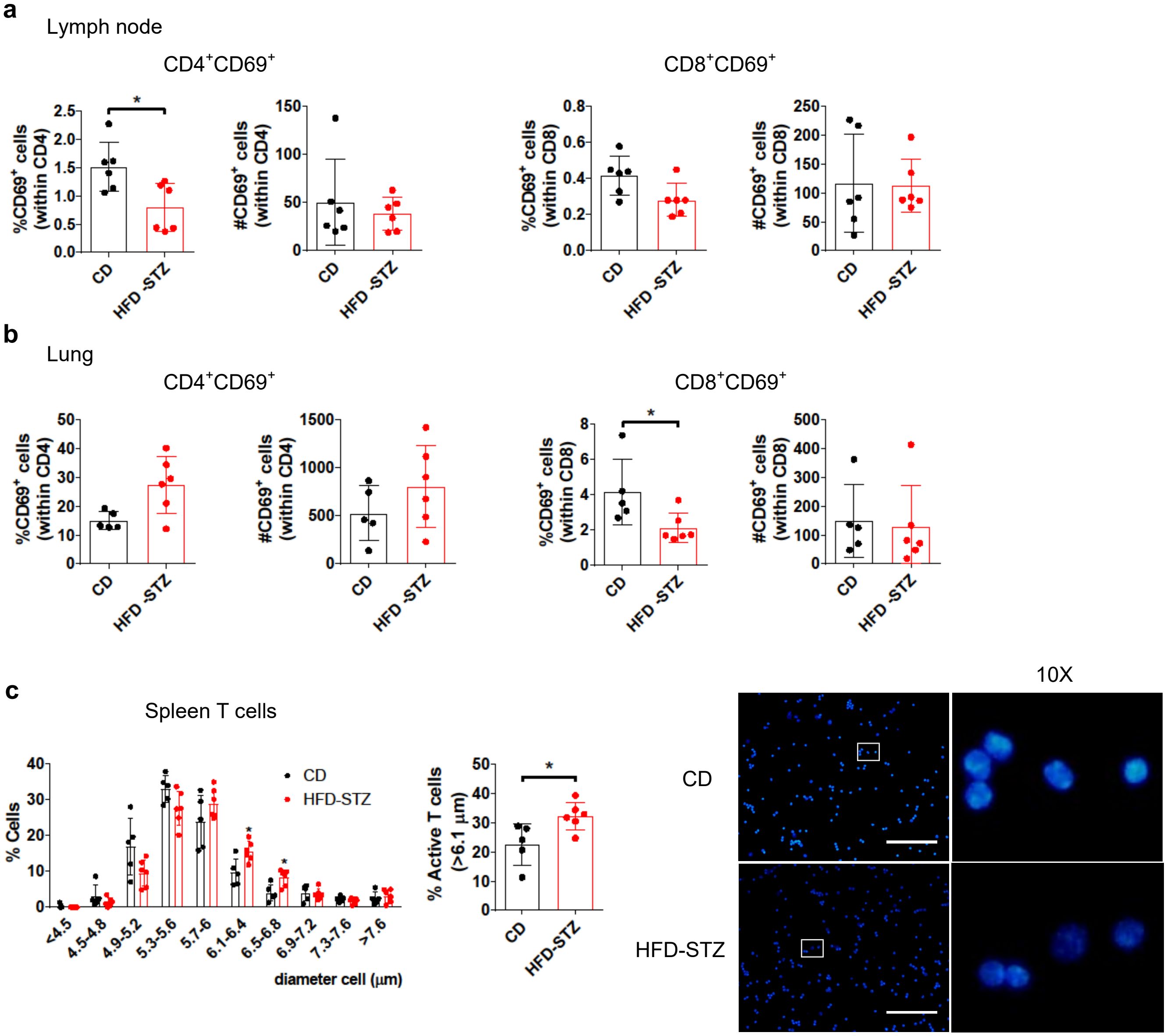

Figure S4. Mice on a high fat diet and treated on streptozotocin have defective innate and adaptive immune responses. Percentage and number of CD69^{+} CD4^{+} and CD8^{+} T cells from control diet mice (*CD*) or mice on a high fat diet and treated with streptozotocin (*HFD-STZ*) in **a** lung-draining lymph nodes (n=6 mice) and **b** lungs (n=5 mice for Cd and n=6 mice for HFD-STZ) 14 days after aerosol infection with ~100 CFU *Mtb* Erdman. **c** Splenic T cells were isolated from uninfected untreated mice or HFD-STZ mice. Nuclei were stained with DAPI and nucleus diameter measured using Image J (*right*). Cells were distributed in bins by nucleus diameter (*left*). Preactivated T cells were classified by nucleus diameter > 6.1 µm (*middle*) (n=5 mice for CD and n=6 mice for HFD-STZ). Scale bar, 100 µm. Data are expressed as mean ± SD. The experiments were repeated at least twice. Statistical analysis was performed by two-tail Student's t-test, *P<0.05 (*p* value from left to right= **a**: 0.0411; **b**: 0.0303; **c**: 0.0173). Source data are provided as Source Data File.

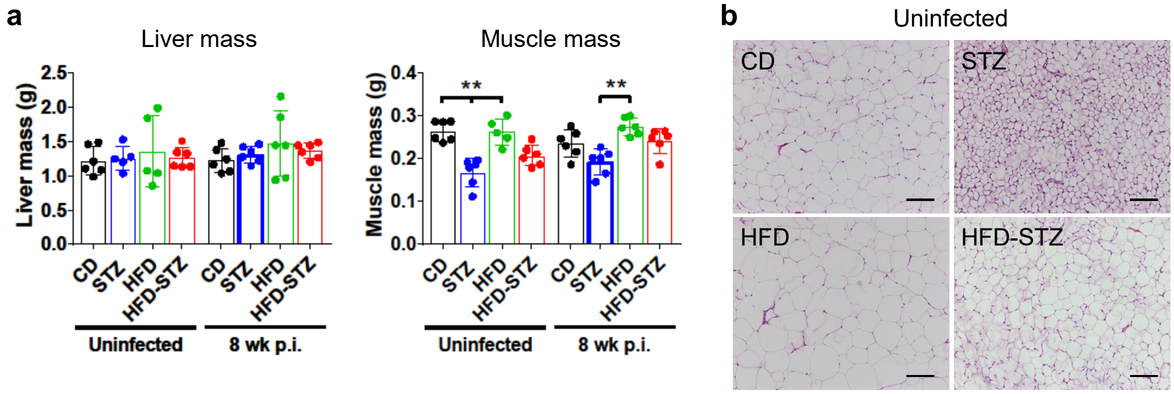

Figure S5. Combination of high fat diet with streptozotocin treatment have increased lipid accumulation in the lungs only during infection. a Liver and skeletal muscle mass of mice on control diet (*CD*) or on high fat diet (*HFD*) mice, treated with streptozotocin (*STZ*) or a combination of both (*HFD-STZ*) (n=5 mice for uninfected STZ and HFD and n=6 for the others). **b** Images of H&E-stained perigonadal adipose tissue (pgWAT) of uninfected mice. Scale bar, 100 µm. Data are expressed as mean ± SD. Statistical analysis was performed by One-Way ANOVA, for untreated or 8 wk p.i. separately, *P<0.05 and **P<0.01 (*p* value from left to right= **a**: 0.0077, 0.0077, 0.0018). Source data are provided as Source Data File.

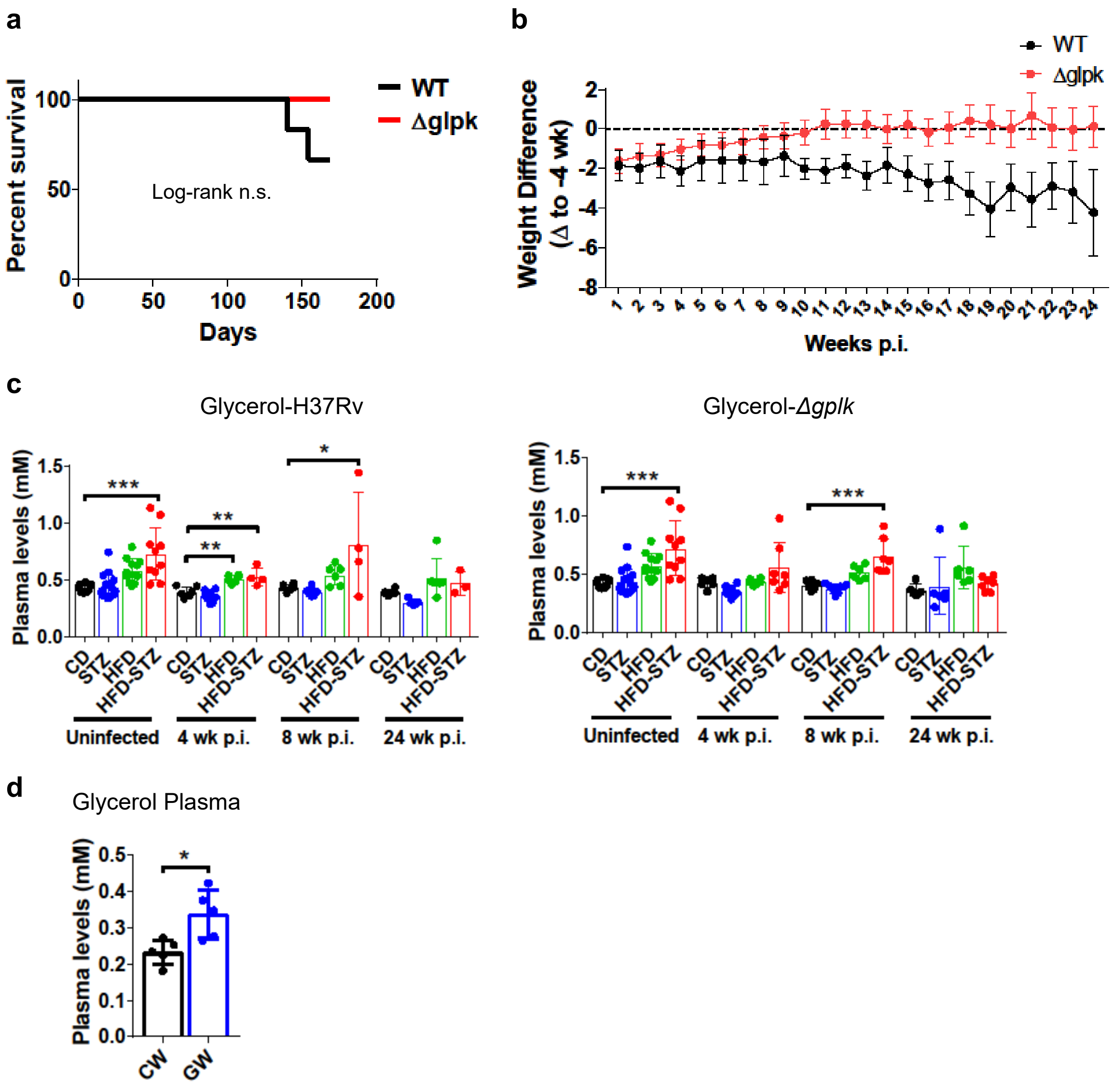

Figure S6. Role of glycerol in the mice on a high fat diet and treated on streptozotocin susceptibility to tuberculosis . Untreated mice (*CD*) or mice treated with a high fat diet (*HFD*) or streptozotocin (*STZ*) or a combination of both (*HFD-STZ*) were aerosol infected with wild type H37Rv or H37Rv lacking glycerol-3-kinase (*ΔglpK*) (~100 CFU) for 24 weeks. **a** Survival curve of HFD-STZ mice infected with both strains (n=8 mice for WT and n=10 mice for *Δ*glpk). Analysis was performed with log-rank Mantel-Cox test. **b** Body weight difference to baseline weight was measured and calculated for HFD-STZ mice infected with H37Rv and *Δglpk* strains (n=8 mice for WT and n=10 mice for *Δ*glpk). **c** Glycerol plasma levels in uninfected mice or mice infected with H37Rv (n= 4 mice for HFD-STZ mice, n=6 mice for CD and STZ and n=8 mice for STZ) or *ΔglpK* (n=6 mice for CD and STZ , n=7 mice for HFD-STZ and n=8 mice for STZ) for 4, 8 and 24 weeks. **d** Glycerol plasma levels in mice that were treated with control water (*CW*) or 5% glycerol water (*GW*) for 3 days (n=5 mice). Data are expressed as mean ± SD. Statistical analysis was performed by One-Way ANOVA for each time point on **c** or two-tail Student's t-test on **d**, *P<0.05, **P<0.01 and ***P<0.001 (*p* value from left to right= **c**: left graph, 0.0001, 0.0044, 0.0035, 0.0175; right graph, 0.0001, 0.0004; **d**: 0.0159). Source data are provided as Source Data File.
