# Supplementary material for: Glycerol contributes to tuberculosis susceptibility in male mice with type 2 diabetes
Source: Nat Commun. 2023 Sep 20;14:5840. doi: 10.1038/s41467-023-41519-9 (PMC10511404; doi:10.1038/s41467-023-41519-9)
Supplement: Supplementary file 3 — Reporting Summary [file 41467_2023_41519_MOESM3_ESM.pdf]

nature portfolio

Corresponding author(s): Hardy Kornfeld

Last updated by author(s): Jul 24, 2023

Reporting Summary

Nature Portfolio wishes to improve the reproducibility of the work that we publish. This form provides structure for consistency and transparency in reporting. For further information on Nature Portfolio policies, see our Editorial Policies and the Editorial Policy Checklist.

Statistics

For all statistical analyses, confirm that the following items are present in the figure legend, table legend, main text, or Methods section.

n/a	Confirmed	
☐	☒	The exact sample size (n) for each experimental group/condition, given as a discrete number and unit of measurement
☐	☒	A statement on whether measurements were taken from distinct samples or whether the same sample was measured repeatedly
☐	☒	The statistical test(s) used AND whether they are one- or two-sided<br>*Only common tests should be described solely by name; describe more complex techniques in the Methods section.*
☒	☐	A description of all covariates tested
☐	☒	A description of any assumptions or corrections, such as tests of normality and adjustment for multiple comparisons
☐	☒	A full description of the statistical parameters including central tendency (e.g. means) or other basic estimates (e.g. regression coefficient) AND variation (e.g. standard deviation) or associated estimates of uncertainty (e.g. confidence intervals)
☐	☒	For null hypothesis testing, the test statistic (e.g. F, t, r) with confidence intervals, effect sizes, degrees of freedom and P value noted<br>*Give P values as exact values whenever suitable.*
☒	☐	For Bayesian analysis, information on the choice of priors and Markov chain Monte Carlo settings
☒	☐	For hierarchical and complex designs, identification of the appropriate level for tests and full reporting of outcomes
☒	☐	Estimates of effect sizes (e.g. Cohen's d, Pearson's r), indicating how they were calculated

Our web collection on statistics for biologists contains articles on many of the points above.

Software and code

Policy information about availability of computer code

Data collection	Data was collected with Microsoft Excel for Microsoft 365 MSO (Version 2304 Build 16.0.16327.20200) 64-bit
Data analysis	Data was analyzed using GraphPad Prism Version 7.04, Image J 1.46r, FlowJo v10.8.1.

For manuscripts utilizing custom algorithms or software that are central to the research but not yet described in published literature, software must be made available to editors and reviewers. We strongly encourage code deposition in a community repository (e.g. GitHub). See the Nature Portfolio guidelines for submitting code & software for further information.

Data

Policy information about availability of data

All manuscripts must include a data availability statement. This statement should provide the following information, where applicable:

- Accession codes, unique identifiers, or web links for publicly available datasets
- A description of any restrictions on data availability
- For clinical datasets or third party data, please ensure that the statement adheres to our policy

No datasets have been generated in this manuscript.

Research involving human participants, their data, or biological material

Policy information about studies with human participants or human data. See also policy information about sex, gender (identity/presentation), and sexual orientation and race, ethnicity and racism.

Reporting on sex and gender	There is no human participants in this study
Reporting on race, ethnicity, or other socially relevant groupings	There is no human participants in this study
Population characteristics	There is no human participants in this study
Recruitment	There is no human participants in this study
Ethics oversight	There is no human participants in this study

Note that full information on the approval of the study protocol must also be provided in the manuscript.

Field-specific reporting

Please select the one below that is the best fit for your research. If you are not sure, read the appropriate sections before making your selection.

[x] Life sciences   [ ] Behavioural & social sciences   [ ] Ecological, evolutionary & environmental sciences

For a reference copy of the document with all sections, see nature.com/documents/nr-reporting-summary-flat.pdf

Life sciences study design

All studies must disclose on these points even when the disclosure is negative.

Sample size	Sample size was determined based on prior published empirical experience where group sizes sufficient to identify statistically significant differences using the case-specific parametric or non-parametric statistical test with correction for multiple comparisons, when indicated, for experimental approaches like those used in the present report. Sample size was n=5-6 mice (variable depending on mice dying from the infection or the treatments), except for survival experiments that were n=8-10 mice
Data exclusions	No data was excluded from the study
Replication	Each experiment was reproduced at least twice and results followed similar trend. No experiment failed.
Randomization	Mice were randomly distributed into the different conditions.
Blinding	Researchers were blinded when lung lesions were quantified.

Reporting for specific materials, systems and methods

We require information from authors about some types of materials, experimental systems and methods used in many studies. Here, indicate whether each material, system or method listed is relevant to your study. If you are not sure if a list item applies to your research, read the appropriate section before selecting a response.

Materials & experimental systems

n/a	Involved in the study	
[ ]	[x]	Antibodies
[x]	[ ]	Eukaryotic cell lines
[x]	[ ]	Palaeontology and archaeology
[ ]	[x]	Animals and other organisms
[x]	[ ]	Clinical data
[x]	[ ]	Dual use research of concern
[x]	[ ]	Plants

Methods

n/a	Involved in the study	
[x]	[ ]	ChIP-seq
[ ]	[x]	Flow cytometry
[x]	[ ]	MRI-based neuroimaging

Antibodies

Antibodies used	For flow cytometry from Biolegend: Zombie Aqua Viability Kit (#423101), CD45 APC-Cy7 (30-F11)(#103115), CD3 PE-Cy7 (17A2)(#100219), CD11c PE (N418)(#117307), CD11b PercP-Cy5.5 (M1/70)(#101227), Ly6C A700 (HK1.4)(#128023), Ly6G FITC (1A8)(#127605), MHC-II Bv421 (M5/114.15.2)(#107631), CD4 FITC (GK1.5)(#100405), CD8 PE (YTS156.77)(#126607), CD3 PercP-Cy5.5 (17A2)(#100217), TNF-α APC (MP6-XT22)(#506307), and IFN-γ Bv421 (XMG1.2)(#505829) used at a 1:100 dilution with the exception

	of MHC-II that was used at a 1:50 dilution. For Western blot from Cell Signaling Technology Inc.: phospho-ACLY (#4331), ACLY (#4332), FASN (#3189), ATGL (#2138), phospho-HSL (#4137), total HSL (#4107) used at a 1:1000 dilution and β-actin (#4970-Lot18) used at a 1:10,000 dilution.
Validation	Validation for zombie Aqua Viability Kit was done by Biolegend using one day old splenocytes. All the flow cytometry antibodies were validated by Biolegend using mouse splenocytes and they have been extensively used in the literature. All the Western Blot antibodies were validated by Cell signaling using mouse cells and they have been extensively used in the literature with knockout cells or mice.

Animals and other research organisms

Policy information about studies involving animals; ARRIVE guidelines recommended for reporting animal research, and Sex and Gender in Research

Laboratory animals	C57BL/6J male mice were used between 6-8 weeks old.
Wild animals	Study did not involve wild animals
Reporting on sex	Only males were used, in previous studies we determined that only 50% of females were responding to streptozotocin treatment. The gender used has been added to the title and the abstract.
Field-collected samples	Study did not involve samples collected in the field.
Ethics oversight	Animal experiments were performed under protocols approved by the Institutional Animal Care and Use Committee (protocol 202100197) and Institutional Biosafety Committee (protocol I-161)

Note that full information on the approval of the study protocol must also be provided in the manuscript.

Flow Cytometry

Plots

Confirm that:

- [x] The axis labels state the marker and fluorochrome used (e.g. CD4-FITC).
- [x] The axis scales are clearly visible. Include numbers along axes only for bottom left plot of group (a 'group' is an analysis of identical markers).
- [x] All plots are contour plots with outliers or pseudocolor plots.
- [x] A numerical value for number of cells or percentage (with statistics) is provided.

Methodology

Sample preparation	Cells were isolated from lungs or spleens. In lungs, cells were dissociated with Collagenase IV for 30 min at 37C and then using miltenyi dissociator in lung program. For spleens, cells were obtained dissociating with a 3 ml syringe plunger on a 70 um strainer. Cells were spun down at 300 g for 5 min and pellet was used for staining.
Instrument	LSRII A-5 with blue, violet, red, UV and yellow-green lasers from BD Biosciences
Software	Data was collected with DIVA software and it was analyzed with FlowJo v10.8.1
Cell population abundance	We did not perform any sorting that required purity check. For flow analysis, in Figure 2d the number of the specified cell populations ranged from 10,000 to 200,000 cells in the different conditions. In Figures S3 and S4 cell numbers ranged from 100 to 1,000 cells in the different conditions.
Gating strategy	Gating for Figure 2d was detailed in Figure S2e. Debris was gated out in SSC-A/FSC-A plot excluding signal that was lower than 20K. Then, singlets were gated with FSC-H/FSC-A. Live cells were gated with SSC-A/Live-Dead taking the cells that were lower than 10^3. From there, CD45+ cells were gated and subpopulations CD3+ were gated. For DC and macrophages CD11c/CD11b plots were drawn, from CD11c+CD11b+ Ly6Chi and Ly6Clow were gated. In each population, MHCII+ cells were gated. From CD11c-CD11b+ cells, Ly6G+ cells were gated. We always took positive cells as higher than 10^3. Gating for Figures S2 and S3 were performed as detailed above. From CD3+ cells, CD4+ and CD8+ cells were gated and each marker was measured as a histogram in each T cell subtype.

- [x] Tick this box to confirm that a figure exemplifying the gating strategy is provided in the Supplementary Information.
